# A Clinical Drug Library Screen Identifies Tosufloxacin as Being Highly Active against *Staphylococcus aureus* Persisters

**DOI:** 10.3390/antibiotics4030329

**Published:** 2015-07-31

**Authors:** Hongxia Niu, Peng Cui, Rebecca Yee, Wanliang Shi, Shuo Zhang, Jie Feng, David Sullivan, Wenhong Zhang, Bingdong Zhu, Ying Zhang

**Affiliations:** 1 Department of Molecular Microbiology and Immunology, Bloomberg School of Public Health, Johns Hopkins University, Baltimore, MD 21205, USA; E-Mails: niuhongxia1985@163.com (H.N.); keanuc@163.com (P.C.); ryee2@jhu.edu (R.Y.); wshi3@jhu.edu (W.S.); shuozhang66@gmail.com (S.Z.); jfeng16@jhu.edu (J.F.); dsulliv7@jhmi.edu (D.S.); 2 Lanzhou Center for Tuberculosis Research and Institute of Pathogenic Biology, School of Basic Medical Sciences, Lanzhou University, Lanzhou 730000, China; E-Mail: bdzhu@lzu.edu.cn; 3 Key Laboratory of Medical Molecular Virology, Department of Infectious Diseases, Huashan Hospital, Shanghai Medical College, Fudan University, Shanghai 200040, China; E-Mail: zhangwenhong@fudan.edu.cn

**Keywords:** *Staphylococcus aureus*, persisters, clinical drug library, tosufloxacin, clinafloxacin

## Abstract

To identify effective compounds that are active against *Staphylococcus aureus* (*S. aureus*) persisters, we screened a clinical drug library consisting of 1524 compounds and identified six drug candidates that had anti-persister activity: tosufloxacin, clinafloxacin, sarafloxacin, doxycycline, thiostrepton, and chlorosalicylanilide. Among them, tosufloxacin had the highest anti-persister activity, which could completely eradicate *S. aureus* persisters within 2 days *in vitro*. Clinafloxacin ranked the second with very few persisters surviving the drug exposure. Interestingly, we found that both tosufloxacin and trovafloxacin that had high activity against persisters contained at the N-1 position the 2,4-difluorophenyl group, which is absent in other less active quinolones and may be associated with the high anti-persister activity. Further studies are needed to evaluate tosufloxacin in animal models and to explain its unique activity against bacterial persisters. Our findings may have implications for improved treatment of persistent bacterial infections.

## 1. Introduction

*Staphylococcus aureus* (*S. aureus*) is a common opportunistic pathogen which can cause infections including, skin infection, pneumonia, bloodstream infection, endocarditis and osteomyelitis. Methicillin-resistant *S. aureus* (MRSA) poses a significant threat in different parts of the world. Besides genetic resistance as in MRSA, *S. aureus* also develops persisters that show phenotypic resistance or tolerance [1] as a fraction of *S. aureus* cells that survived lethal antibiotic treatment [2,3,4]. In addition, it has been demonstrated that persisters of *S. aureus* survived antibiotic treatment in prosthetic biofilm infection in the mouse model [5]. Clinically, bacteremia and endocarditis caused by *S. aureus* can be difficult to cure with current antibiotics that are mainly active against growing bacteria but not against non-growing persister bacteria. It has been proposed to target both growing bacteria and non-growing persisters for more effective treatment of persistent infections [6]. Here, as part of the efforts to identify effective drugs targeting bacterial persisters, we screened a clinical drug library on stationary-phase *S. aureus* persisters.

## 2. Results and Discussion

**Figure 1:**
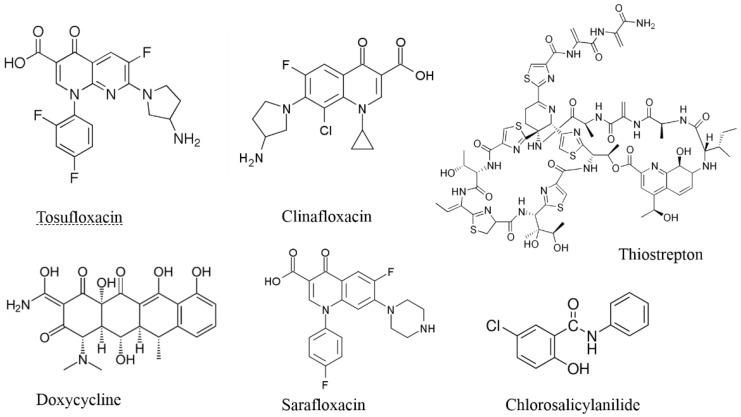
Structures of the six drug candidates with activity against *S. aureus* persisters.

Of the 1524 drugs in the clinical drug library tested, six compounds (tosufloxacin, clinafloxacin, sarafloxacin, doxycycline, thiostrepton, and chlorosalicylanilide) produced no colonies on LB plates. Based on the results of the primary screen, we selected these drug candidates (50 μM) for rescreens using the same method as above to rank the relative activity of these drugs against *S. aureus* persisters over time. The bacterial survival was monitored daily for 4 days in drug exposure assay by transferring the bacterial suspension onto TSB agar plates, respectively. Among them, tosufloxacin produced no colonies on TSB plates after 1 day drug exposure; clinafloxacin and thiostrepton produced no colonies on TSB plates after 2 days drug exposure; doxycycline and chlorosalicylanilide produced no colonies on TSB plates after 3 days drug exposure. Sarafloxacin produced no colonies on TSB plates after 4 days drug exposure (Table 1). The MICs and MBCs of the six drug candidates are shown in Table 1. The structures of the six drug candidates that showed anti-persister activity are shown in Figure 1. Tosufloxacin, clinafloxacin and sarafloxacin are quinolone drugs, which exert their antibacterial effect by preventing bacterial DNA from unwinding and replicating [7]. Doxycycline and thiostrepton interfere with bacterial protein synthesis by inhibiting prolongation of peptide chains when binding to 30S or 50S ribosomal subunits [8,9]. Chlorosalicylanilide is a brominated derivative of salicylanilide, and its antibacterial effect may be mediated by inhibiting cellular respiration and energy production.

**Table 1:** Activity of six drug candidates (50 μM) selected from the clinical drug library against stationary-phase *S. aureus* persisters.

Drugs (50 μM)	MIC (μM)	MBC (μM)	Bacterial survival at different times of drug exposure ^a^			
			Day 1	Day 2	Day 3	Day 4
Tosufloxacin	<0.3	0.3	–	–	–	–
Clinafloxacin	0.3	0.3	+	–	–	–
Thiostrepton	2.5	–	+	–	–	–
Doxycycline	<0.3	0.6	+	+	–	–
Chlorosalicylanilide	>10	–	+	+	–	–
Sarafloxacin	0.3	–	+	+	+	–
Drug-free Control	-	–	+	+	+	+

^a^ Stationary-phase *S. aureus* bacteria that survived ofloxacin treatment were used as persisters which were then treated with different drug candidates for 1, 2, 3 or 4 days, and the viability of the bacteria was detected by transfer on the LB plates using a 96-pin replicator; “–” No colonies grew on LB plates after drug exposure; “+” Indicates obvious colonies grew on LB plates after drug exposure.

**Figure 2:**
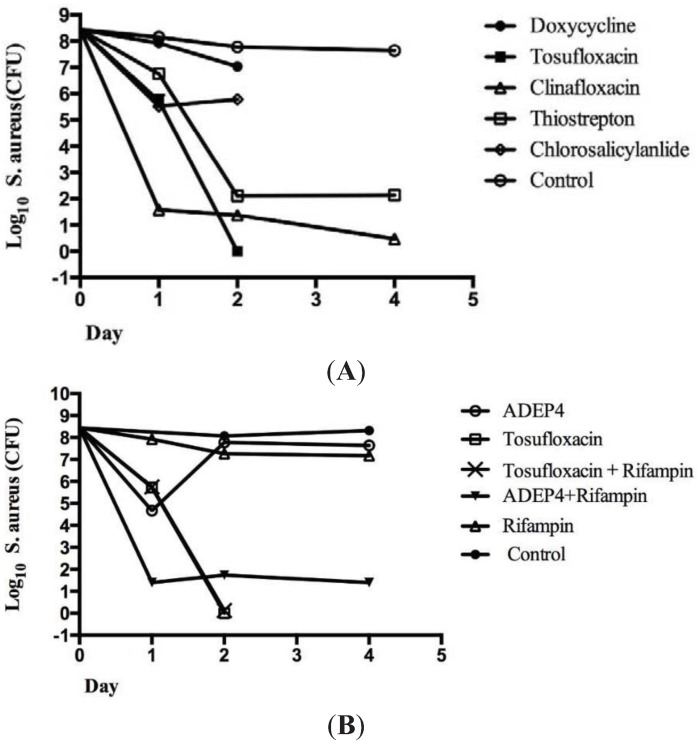
Colony counts to determine the relative activity of the drug candidates and in comparison to known agents active against *S. aureus* persisters. The *S. aureus* stationary phase culture was treated with ofloxacin (10 μg/mL) for four hours to enrich persisters. Then, the surviving persisters from the treatment were subjected to drug exposure with different antibiotics (50 μM) as described in the text. The viability of the bacteria was determined by colony forming unit (CFU) count 1 to 4 days after drug exposure. (**A**) Tosufloxacin (50 μM) was the most active among the six drugs, and completely eliminated all persisters after 2 days; (**B**) acyldepsipeptide (ADEP4) + rifampin (RIF) could not completely eradicate *S. aureus* persisters even after 4 days. The final concentrations of ADEP4 and RIF were 5 μg/mL and 0.4 μg/mL, respectively.

To more accurately rank the six effective drug candidates identified using the 96-well plate method, colony forming unit (CFU) count was also performed after 1 to 4 day drug exposure of persister bacteria in MOPS buffer in Eppendorf tubes. Tosufloxacin had the highest activity against *S. aureus* persisters, eradicating all the persisters within 2 days (Figure 2A). Clinafloxacin was the second highest active drug candidate followed by thiostrepton, decreasing the CFU of the *S. aureus* persisters to 10 and 100 respectively after 4 day drug exposure. However, chlorosalicylanilide and doxycycline had limited activity against *S. aureus* persisters and the persister level was still at more than 10^6^ CFU/mL after drug exposure (Figure 2A). 

**Figure 3:**
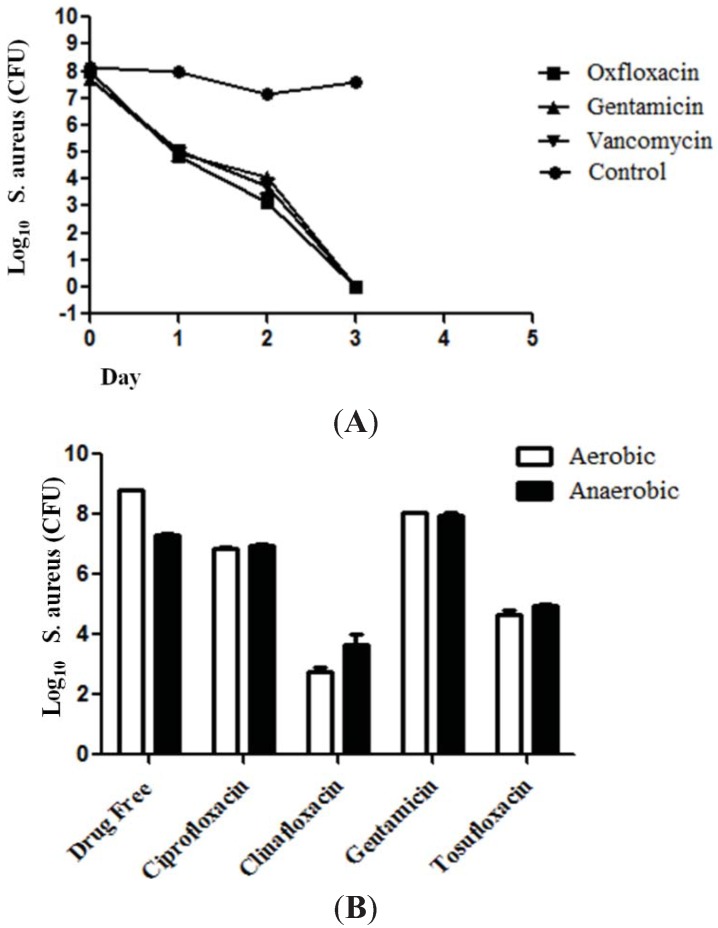
The effect of tosufloxacin against *S. aureus* persisters that survived from different cidal antibiotic treatments and comparison of the activity of antibiotics in aerobic and anaerobic environments. (**A**) *S. aureus* persisters enriched by ofloxacin, gentamycin or vancomycin were similarly killed by tosufloxacin (50 μM). The *S. aureus* stationary phase culture was treated with ofloxacin (10 μg/mL), gentamycin (10 μg/mL) or vancomycin (10 μg/mL) for four hours to enrich persisters. Then, the surviving persisters from the treatment were subjected to drug exposure with tosufloxacin (50 μM); (**B**) The activities of antibiotics were comparable in aerobic and anaerobic environments. The *S. aureus* stationary phase culture was exposed to 20 μM of the respective drugs and incubated for 3 days at 37 °C (aerobically and anaerobically) when the CFU was determined.

It has been reported that the combination of acyldepsipeptide (ADEP4) and rifampin produced complete eradication of *S. aureus* biofilms *in vitro* and *in vivo* [10]. ADEP4 when used alone had limited activity and that is the reason for the use of ADEP4 and RIF combination in this study for comparison with tosufloxacin. Therefore, we compared tosufloxacin identified from the clinical drug library with ADEP4 + RIF for their activities against *S. aureus* persisters *in vitro*. We found that while ADEP4+RIF had an initial rapid killing effect in Day 1 the combination failed to completely eradicate all *S. aureus* persisters and there were still about 100 residual persisters left even after 4 day drug exposure (Figure 2B). In contrast, despite a slow killing effect initially at Day 1, tosufloxacin completely eradicated all *S. aureus* persisters in 2 days with no persisters surviving (Figure 2B). We also found that *S. aureus* persisters derived from other cidal antibiotics gentamicin and vancomycin could similarly be killed by tosufloxacin as those derived from ofloxacin pretreatment (Figure 3A), and the activity of antibiotics against *S. aureus* persisters was comparable in aerobic and anaerobic conditions (Figure 3B).

The presence of persisters makes the treatment of many bacterial infections such as urinary tract infections, prosthetic biofilm infections very challenging. The ability to efficiently eradicate the infection without relapse will help reduce the spread of drug resistance. Since clinical drugs have relatively clear safety and pharmacokinetic profiles in humans, studies examining whether existing clinical drugs could effectively eliminate *S. aureus* persisters represent a rapid and efficient approach to addressing this problem. In this study, we found that tosufloxacin had the best activity against *S. aureus* persisters *in vitro* among the six active drug candidates (Table 1, Figure 2). It is worth noting that we recently found that tosufloxacin also had high activity against *Escherichia coli* persisters [11]. Therefore, tosufloxacin may be a truly effective drug candidate against persisters of both Gram-positive and Gram-negative bacteria. Further studies are required to evaluate tosufloxacin and its combination with other antibiotics in animal models of persistent infections and to explain its unique activity against bacterial persisters.

**Figure 4:**
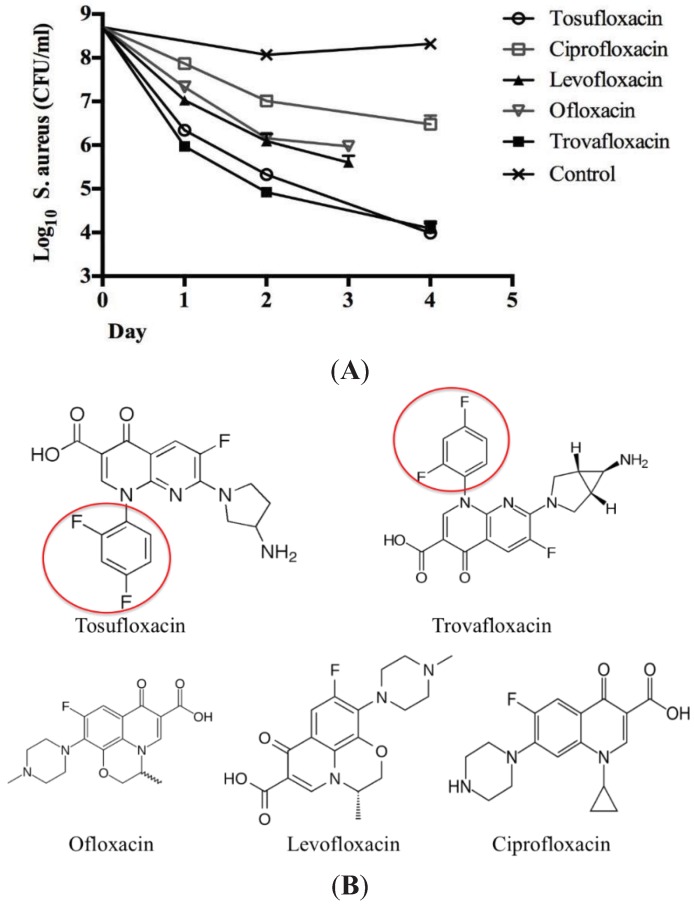
Comparison of the structure and activity of tosufloxacin with the other quinolone drugs. (**A**) The activity of quinolone drugs (10 μM) against *S. aureus* persisters. *S. aureus* stationary phase culture was treated with ofloxacin (10 μg/mL) for 4 h to enrich persisters followed by treatment with different quinolone antibiotics. The bacterial viability was determined at different times by CFU count; (**B**) Structures of tosufloxacin and the other quinolone antibiotics. Tosufloxacin and trovafloxacin that have high activity against persisters both contain the 2,4-difluorophenyl group at the N-1 position, which is highlighted by the red circle and is absent in other less active quinolones.

Tosufloxacin is a fluoroquinolone drug characterized by the 2,4-difluorophenyl and 3-amino-1-pyrrolidinyl groups at the quinolone nucleus. It has been reported that compounds with a 2,4-difluorophenyl group at the N-1 position in the quinolone nucleus exhibited good bactericidal activities [12]. Here, we compared tosufloxacin and trovafloxacin which both contain the same 2,4-difluorophenyl group at the N-1 position with other quinolone drugs (ofloxacin, levofloxacin, ciprofloxacin) for their ability to kill *S. aureus* persisters. We found that indeed both tosufloxacin and trovafloxacin showed higher anti-persister activity than other fluoroquinolones without the 2,4-difluorophenyl group at the N-1 position (Figure 4A,B). Therefore, we speculate that the high activity of tosufloxacin and trovafloxacin against *S. aureus* persisters may be due to the 2,4-difluorophenyl group at the N-1 position. Future structure activity studies are needed to test this possibility.

## 3. Experimental Section

*S. aureus* Newman, a strain that was originally isolated from a patient suffering from osteomyelitis [13], was cultured in Tryptic soy broth (TSB) medium overnight to stationary phase. Then, the overnight culture was treated with ofloxacin (10 μg/mL) for 4 h to kill growing bacteria and enrich persisters. Finally, the persisters that survived the ofloxacin treatment were washed twice and resuspended in 3-(*N*-morpholino) propanesulfonic acid (MOPS) buffer and 100 μL bacterial suspension was transferred to 96-well microplates for drug screens. The clinical drug library consisting of 1524 pharmacologically active compounds, which were either FDA-approved drugs or drugs approved for use abroad, was prepared as 10 mM stock solutions and 1 mM working stock solution in dimethyl sulfoxide (DMSO) and was arrayed in a total of 24 96-well plates [14], leaving the first and last columns in each plate for controls. To qualitatively determine the effect of clinical drugs on *S. aureus* persisters, each compound from the working stock solution (5 μL) was added to the persister cell suspension to achieve a final drug concentration of 50 μM in the drug screen. The plates were sealed and placed in a 37 °C incubator for 5 days without shaking. After 5 days of drug exposure, a 96-pin replicator was used to transfer the bacterial suspension onto TSB agar plates, to monitor the bacterial survival after drug exposure.

To more quantitatively validate the active drug candidates, colony forming unit (CFU) count was performed. The surviving persisters from the ofloxacin treatment (10 μg/mL) were dispensed into 1.5 mL Eppendorf tubes in 1 mL MOPS buffer followed by addition of drugs at a final concentration of 50 μM. After 1 to 4 day drug exposure, 100 μL bacterial suspension was removed followed by washing in PBS. Serial dilutions were spotted onto TSB agar plates to get CFU count after incubation overnight at 37 °C.

## 4. Conclusions

In conclusion, we identified six drug candidates that have activity against *S. aureus* persisters with tosufloxacin having the highest anti-persister activity. Both tosufloxacin and trovafloxacin were highly active against *S. aureus* persisters which may be attributable to their common 2,4-difluorophenyl group. Further studies are needed to evaluate tosufloxacin in animal models and to explain its unique activity against bacterial persisters. These findings may have implications for improved treatment of persistent bacterial infections.
